# Vangl2, a Core Component of the WNT/PCP Pathway, Regulates Adult Hippocampal Neurogenesis and Age-Related Decline in Cognitive Flexibility

**DOI:** 10.3389/fnagi.2022.844255

**Published:** 2022-03-09

**Authors:** Muriel Koehl, Elodie Ladevèze, Mireille Montcouquiol, Djoher Nora Abrous

**Affiliations:** ^1^Univ. Bordeaux, INSERM, Magendie, U1215, Neurogenesis and Pathophysiology group, Bordeaux, France; ^2^Univ. Bordeaux, INSERM, Magendie, U1215, Planar Polarity and Plasticity Group, Bordeaux, France

**Keywords:** hippocampus, Vangl2, planar cell polarity, memory, forgetting, flexibility, interferences, adult neurogenesis

## Abstract

Decline in episodic memory is one of the hallmarks of aging and represents one of the most important health problems facing Western societies. A key structure in episodic memory is the hippocampal formation and the dentate gyrus in particular, as the continuous production of new dentate granule neurons in this brain region was found to play a crucial role in memory and age-related decline in memory. As such, understanding the molecular processes that regulate the relationship between adult neurogenesis and aging of memory function holds great therapeutic potential. Recently, we found that Vang-Gogh like 2 (Vangl2), a core component of the Planar Cell Polarity (PCP) signaling pathway, is enriched in the dentate gyrus of adult mice. In this context, we sought to evaluate the involvement of this member of the Wnt/PCP pathway in both adult neurogenesis and memory abilities in adult and middle-aged mice. Using a heterozygous mouse model carrying a dominant-negative mutation in the Vangl2 gene, called Looptail (Vangl2^Lp^), we show that alteration in Vangl2 expression decreases the survival of adult-born granule cells and advances the onset of a decrease in cognitive flexibility. The inability of mutant mice to erase old irrelevant information to the benefit of new relevant ones highlights a key role of Vangl2 in interference-based forgetting. Taken together, our findings show that Vangl2 activity may constitute an interesting target to prevent age-related decline in hippocampal plasticity and memory.

## Introduction

Cognitive decline associated with aging represents one of the most important health problems facing Western societies. The memory for specific events, the so-called episodic memory (Eichenbaum, 2000), is particularly sensitive to the effect of aging and declines from early middle age (Nyberg and Pudas, 2019). The different processes involved in episodic memory formation, i.e., the ability to encode and retrieve past experiences and to use the learned information in a novel condition, rely upon the hippocampal formation (Eichenbaum et al., 1990; Bunsey and Eichenbaum, 1996), and impairment of its integrity is considered a key phenomenon in the appearance of age-related memory deficits (Gonzalez-Escamilla et al., 2018; Mota et al., 2019).

One key hippocampal sub-structure involved in episodic memory is the dentate gyrus (DG), one of the two brain regions in the adult mammalian brain that retains the capability to produce new neurons throughout adult life. Briefly, the radial glia-like neural stem cells (NSCs) located in the subgranular zone (SGZ), at the interface between the hilus and the granule cell layer (GCL), leave quiescence to proliferate, and through asymmetrical division generate transient amplifying neural progenitor cells (NPCs). These NPCs have the potential to differentiate into neuroblasts and dentate granule neurons (DGN) that mature over several weeks and integrate the existing hippocampal circuitry in order to maintain critical hippocampal functions throughout adulthood (Abrous et al., 2005; Christian et al., 2020). More specifically, rodent studies have shown that adult hippocampal neurogenesis is primarily involved in encoding and retrieving similar events (a process called pattern separation), as well as in using a previously learned information in a flexible way, an ability that is required to navigate through space (Dupret et al., 2008; Baptista and Andrade, 2018). In the context of aging, we have previously uncovered the existence of a link between the rate of new neurons production in the aging DG and the memory abilities of senescent animals: preserved memory functions are associated with the maintenance of a relatively high neurogenesis level whereas memory deficits are linked to exhaustion of neurogenesis after learning (Drapeau et al., 2003). More recently, we have deepened this link by showing that the maintenance of healthy newborn neurons in the course of aging provides resilience to cognitive aging (Montaron et al., 2020).

In this context, a better understanding of the molecular processes that regulate the relationship between adult neurogenesis and cognitive aging is important, not only to provide insight into the mechanisms involved in the resilience/vulnerability to cognitive aging but also to develop new preventing/curing strategies. To do so, we focused on Wnt signaling, because it is compromised in the aging brain (Palomer et al., 2019) and its members were found to regulate different aspects of adult hippocampal neurogenesis (e.g., Lie et al., 2005; Wexler et al., 2009; Varela-Nallar and Inestrosa, 2013; Mardones et al., 2016; Arredondo et al., 2020). Recently, we showed that the 4-transmembrane protein Van-Gogh-like 2 (Vangl2) a member of the Wnt core PCP pathway (Montcouquiol et al., 2006; Montcouquiol and Kelley, 2020), is enriched in young postmitotic neurons of the neurogenic zone of the adult DG (Robert et al., 2020), which led us to investigate the *in vivo* effects of mutation in the Vangl2 gene on adult hippocampal neurogenesis and spatial navigation in the course of aging. We used for this study a mouse model carrying a spontaneous dominant-negative mutation for *Vangl2* (called Loop-tail or Vangl2^Lp^). We show here that mutation of the Vangl2 gene decreases neurogenesis by reducing newborn cells’ survival. These deficits are associated in adult mice with a dampening in memory flexibility and additional deficits in handling memory interferences and spatial navigation at middle age. Altogether, this study is the first to show that alterations in the Wnt/PCP signaling pathway is a key factor in accelerating age-related alterations in spatial components of episodic-like memory in association with an impairment of adult neurogenesis.

## Materials and Methods

### Animals

Looptail mutant mice of the LPT/Le stock were originally obtained from Jackson Laboratories (Laboratory stock No. 000220). These mice carry a dominant-negative point mutation at position 464 that causes a serine to asparagine amino acid change resulting in loss of function (Kibar et al., 2001; Murdoch, 2001; Yin et al., 2012). The mouse colony was maintained at Neurocentre Magendie under standard conditions by heterozygous intercross. Only heterozygous *Vangl2^Lp/+^* and their WT littermates *Vangl2^+/+^* were used in this study as homozygous fetuses die perinatally. Heterozygous mice were initially identified by the presence of a looped tail, and biopsies of fixed brains were later analyzed to confirm genotype. Genotyping was performed by direct sequencing of PCR-amplified products generated with the following primers: reverse CTGCAGCCGCATGACGAACT, and forward CCTTCCTGGAGCGATATTTG, designed to flank the point mutation. PCR was performed in 20 μl reaction volumes, using GoTaq G2 Hot Start Green Master Mix (Promega), and 0.2 μM of each primer. PCR products were sequenced with the forward primer by the Sanger method.

**Figure 1:**
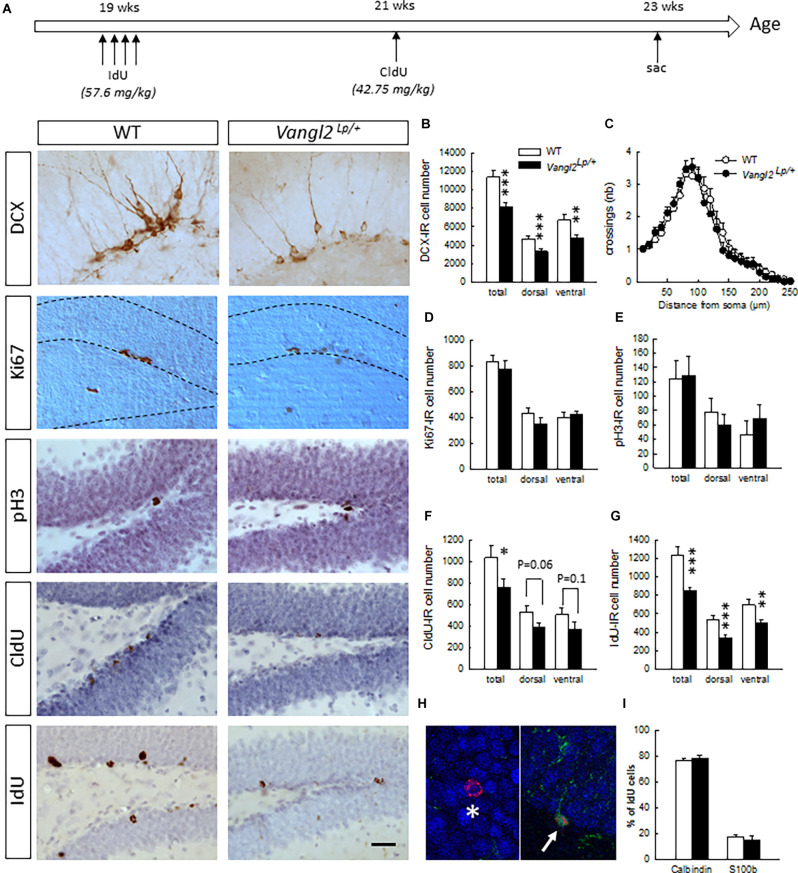
*Vangl2^lp^* mutation decreases survival of adult-born cells in the hippocampal neurogenic niche. **(A)** Timeline of the experiment and representative images of immunostainings in WT and Vangl2^Lp/+^ mice. **(B)** Number of immature neurons (DCX-IR). **(C)** Sholl morphometric analysis of DCX-IR cells. **(D)** Number of Ki67-IR proliferating cells. **(E)** Number of pH3-IR proliferative cells. **(F)** Number of 2-week-old surviving cells (CldU-IR). **(G)** Number of 4-week-old surviving cells (IdU-IR). **(H)** Illustration of a 4-week-old newborn neuron (IdU-Calbindin-IR cell, white star) and a 4-week-old newborn glial cell (IdU-S100-IR cell, white arrow). **(I)** Percentages of neuronal and glial differentiation of 4-week-old IdU-IR cells. White bars, WT *n* = 6–7 mice ; Black bars, Vangl2^Lp/+^ *n* = 8–9 mice; white dots: dendrites WT *n* = 42 neurons (23 in the dorsal and 19 in the ventral DG); black dots, dendrites Vangl2^Lp/+^ *n* = 32 neurons (18 in the dorsal and 14 in the ventral DG). *p < 0.05; **p < 0.01; ***p < 0.001. Scale bar = 50 μm.

**Figure 2:**
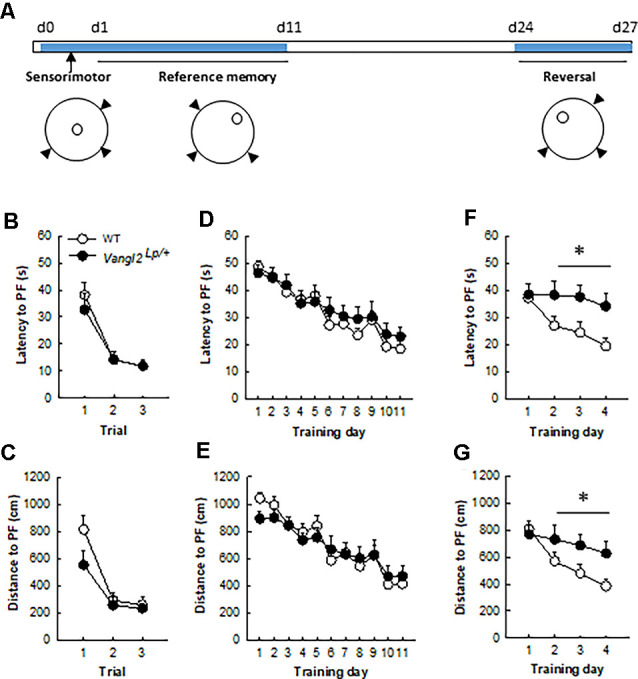
*Vangl2^lp^* mutation leads to deficits in memory flexibility in 4-month-old mice. **(A)** Timeline of the experiment. **(B)** Latency to find a cued platform. **(C)** Distance to find a cued platform. **(D)** Latency to find a hidden platform in a reference memory protocol. **(E)** Distance to find a hidden platform in a reference memory protocol. **(F)** Latency to find a hidden platform in a reversal protocol. **(G)** Distance to find a hidden platform in a reversal protocol. White dots, WT *n* = 20 mice; Black dots, Vangl2^Lp/+^ *n* = 12 mice. *p < 0.05.

**Figure 3:**
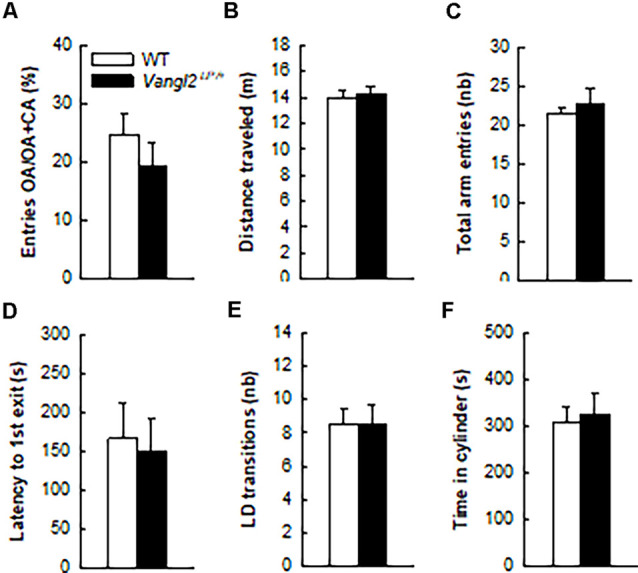
*Vangl2^lp^* mutation does not affect anxiety-related phenotype. Anxiety-like responses were measured in the elevated plus maze **(A–C)** and in the light/dark emergence task **(D–F)**. White bars, WT *n* = 21 mice; Black bars, Vangl2^Lp/+^ *n* = 16 mice.

**Figure 4:**
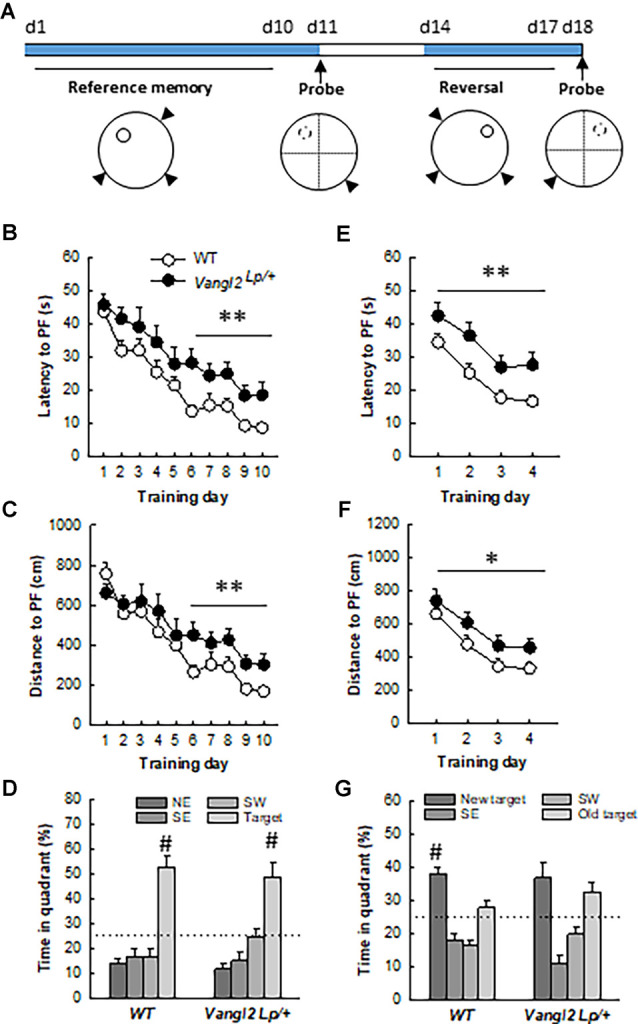
*Vangl2^lp^* mutation increases vulnerability to age-related decline in memory.** (A)** Timeline of the experiment. **(B)** Latency to find a hidden platform in a reference memory protocol. **(C)** Distance to find a hidden platform in a reference memory protocol. **(D)** Percentage of time spent in each quadrant of the water maze during the probe test. **(E)** Latency to find a hidden platform in a reversal protocol. **(F)** Distance to find a hidden platform in a reversal protocol. **(G)** Percentage of time spent in each quadrant of the water maze during the second probe test. White dots, WT *n* = 18 mice; Black dots, Vangl2^Lp/+^ *n* = 11 mice. WT different from Vangl2^Lp/+^ at *p < 0.05, **p < 0.01. ^#^Different from all other positions, NK *p* < 0.01 for all comparisons.

Animals were housed in standard plastic rodent cages and maintained on a 12 h light/dark cycle (light off at 8 p.m) with free access to water and food. Only male littermates, housed in individual cages at least 2 weeks before any intervention, were used in all experiments. Two batches of mice were used in order to study on one hand adult neurogenesis (Batch 1, Figure 1), and on the other hand behavioral capabilities (batch 2, Figures 2–4). In both batches of mice a significant decrease in body weight was observed in *Vangl2^Lp/+^* mice compared to their WT littermates (Batch 1, 4 months old mice: WT = 38.14 ± 0.82g, *Vangl2^Lp/+^* = 29.67 ± 0.64g, *t*_14_ = −8.2 *p* < 0.001; Batch 2, 13 months old mice: WT = 39.62 ± 0.38g, *Vangl2^Lp/+^* = 36.04 ± 0.67g, *t*_35_ = −3.68 *p* < 0.001). No other gross anatomical defect could be observed. All procedures and experimental protocols were approved by the Animal Care Committee of Bordeaux (CEEA50) and were conducted in accordance with the European community’s council directive of 24 November 1986 (86/609/ECC).

### Thymidine Analog Injections

Naïve 5 month-old mice received a daily i.p. injection of IodoDeoxyUridine (IdU; 57.6 mg/kg dissolved in 1N NH_4_OH/NaCl, Sigma) for 4 consecutive days. Two weeks later, the same mice were injected once i.p. with Chlorodeoxyuridine (CldU; 42.75 mg/kg dissolved in saline, Sigma). Mice were then left undisturbed for 2 weeks upon which they were sacrificed to analyze neurogenesis (Figure 1A).

### Immunohistochemistry

Mice were anesthetized with pentobarbital (100 mg/kg) and transcardially perfused with 30 ml of PBS, pH = 7.3 and 30 ml of 4% paraformaldehyde in 0.1 M PB, pH = 7.3. Brains were extracted and kept in paraformaldehyde until vibratome sectioning. Sections were sequentially collected in 10 sets of serial coronal slices of 40 μm thickness and stored in PBS-azide (0.02%) at 4°C until processing.

Immunoperoxydase detection of each neurogenesis marker was performed on one-in-10 free floating sections. The following primary antibodies were used: mouse anti-BrdU (1/500, BD Biosciences, used for IdU detection), rat anti-BrdU (1/1000, Accurate, used for CldU detection), rabbit anti-Ki67 (1/1000, Novocastra), rabbit anti-pH3 (1/1000, Merck Millipore), goat anti-DCX (1/1000, Santa Cruz Biotechnology). Bound antibodies were visualized with horse anti-mouse (1/200, Dako), goat anti-rat (1/1000, Dako), goat anti-rabbit (1/200, Dako), and donkey anti-goat (1/500, Jackson) biotin-labeled secondary antibodies. Briefly, after quenching endogenous tissue peroxidases with 0.3% H_2_O_2_ in methanol for 30 min, sections were incubated for 45 min in PBS-triton X-100 (0.3%) in the presence of 3–5% of normal serum of goat (for CldU, Ki67, and pH3 stainings), horse (for IdU staining), or donkey (for DCX staining) in order to permeabilize membranes and reduce non-specific binding. Sections were then incubated for 72 h at 4°C with primary antibodies. For IdU and CldU stainings, an unmasking step with 2N HCl (30 min at 37°C) was applied prior to primary antibody incubation. Secondary antibody incubation lasted 2 h and was followed by avidin-biotin complexes for signal amplification (ABC kit, Dako). Immunoreactivities were visualized using 3,3’-diaminobenzidine as a chromogen. Finally, IdU and CldU stained sections were counterstained with hematoxylin in order to analyze DG and OB volumes.

For phenotyping newborn cells, triple immunofluorescent IdU-Calbindin-S100 labeling was performed; floating sections were first treated with 2N HCl (30 min at 37°C), incubated for 45 min in PBS containing 5% goat normal serum and 0.3% triton-X-100, followed by 72 h of incubation with a mixture of mouse anti-IdU (1/1000; BD biosciences), guinea pig anti-calbindin (1/300, Synaptic System) and rabbit anti-S100 (1/1000, Sigma) antibodies in PBS-Triton-X-100. Immunoreactivities were revealed with Alexa 568 goat anti-mouse (1/1000, Invitrogen), Alexa 647 goat anti-guinea-pig (1/1000, Jackson), and Alexa 488 goat anti-rabbit (1/1000, Invitrogen) secondary antibodies. Sections were mounted on glass slides and coverslipped with polyvinyl alcohol mounting medium with 1, 4-diazabicyclo[2.2.2] octane (PVA-DABCO).

### Stereological Analysis

An unbiased estimate of IdU, CldU, Ki67, pH3, and DCX -positive cell number in the dentate gyrus was calculated using every 10th section along the rostro-caudal axis. Exhaustive counting in the left hemisphere under 630x or 1000x magnification was used for all markers. Dorsal sections of the DG were defined as sections with coordinates extending from −1 to −2.4 mm antero-posterior from Bregma, and ventral sections included sections from −2.4 to −3.9 from Bregma. For the olfactory bulb (OB), 50 × 50 mm counting frames at evenly spaced intervals of 300 × 300 mm, with an exclusion guard zone of 2 mm, was used to evaluate the number of IdU-IR cells (Stereo Investigator software, MicroBrightField, VT, USA). Results are expressed in the DG as the total number of cells in both hemispheres, and in the OB as cell volumetric density (number of cells per mm^3^).

### Dendrite Analysis

Dendritic processes of DCX-IR cells were traced using a computer-controlled microscope-based system (Leica DM5000; 100× oil-immersion objective) with a software (Neurolucida^®^ software; MicroBrigthField Bioscience; Williston, Vermont) that provides neuron tracing tools to trace from a live camera image (QImaging). Only neurons showing tertiary processes with a vertically-oriented cell body, complete staining of the dendritic tree, and that were not overlapping with neighboring cells were selected for analysis. Soma size, number of branch points, and total length of the immunopositive dendritic tree were measured. A Sholl analysis was further conducted on the reconstructed neurons.

### Phenotype Analysis

The percentage of IdU-labeled cells co-expressing calbindin or S100 was determined throughout the DG. For each animal, IdU-positive cells were randomly selected and analyzed for coexpression with calbindin or S100. Using a confocal microscope (DMR TCS SP2; Leica Microsystems) equipped with a 63 PL APO oil objective, an argon laser (488 nm), a green helium-neon laser (543 nm), and a red helium-neon laser (633 nm) each selected IdU-positive cell was analyzed in its entire z-axis using 1 μm intervals.

### Behavioral Testing

A separate batch of mice was tested for behavioral capabilities. Naïve 3 month-old mice were first tested for anxiety-related behavior. One month and 9 months later (at 4 and 12 months of age), their learning and memory abilities were measured in the Morris water maze.

### Measurement of Anxiety-Related Behaviors

*The Elevated Plus Maze (EPM)* was conducted in an apparatus composed of transparent Plexiglas with two open (45 × 5 cm) and two enclosed (45 × 5 × 17 cm) arms that extended from a common central squared platform (5 × 5 cm). The floor of the maze was covered with black Makrolon and was elevated 116 cm above the floor. A small raised lip (0.5 cm) around the edges of the open arms prevented animals from slipping off. The test session began with the mouse individually placed on the center square facing an open arm. Animals were allowed to freely explore the maze for 5 min under mildly anxiogenic conditions (dim light of 45 lux using a halogen lamp). A camera connected to a computer was utilized to track the mouse path during the entire session (^©^VideoTrack, Viewpoint). Automatic path analysis measured the time spent and the total number of entries into the open and closed arms.

*The light/dark emergence test (LD)* was conducted 2 days later in an open-field (square arena of 50 × 50 cm closed by a wall of 50 cm high and made in white PVC) brightly lit (~400 lux) containing a cylinder (10 cm deep, 6.5 cm in diameter, dark gray PVC) located length-wise along one wall, with the open end 10 cm from the corner. Mice were placed into the cylinder and their behavior recorded for 15 min with a video tracking system (^©^VideoTrack, Viewpoint). Initial latency to emerge from the cylinder, defined as placement of all four paws into the open field, as well as the total number of entries into the cylinder and the total time spent inside the cylinder were analyzed.

### Measurement of Learning and Memory Abilities in the Water Maze

Testing took place in a circular swimming pool (150 cm in diameter) located in a room with various distal cues and filled with water maintained at room temperature (19–20°C) and made opaque by the addition of a nontoxic cosmetic adjuvant. Data were collected using a video camera fixed to the ceiling of the room and connected to a computerized tracking system (Videotrack, Viewpoint) located in an adjacent room that also contained the individual home cages of the mice during testing. The tracking system allowed the calculation of escape latency and path length covered by a mouse until it finds the platform.

#### Pre-training

Mice received a two-step pre-training session. First, they were placed for 15 s onto an escape platform (14 cm diameter, 1 cm above water surface) located in the center of the pool upon which they were released into the water for 30 s and guided to the platform where they had to stay for 15 s. This step was repeated until all mice were able to climb and stay on the platform for 15 s (usually one trial). Once all mice had acquired this step, the platform was lowered below water surface (1–1.5 cm) and the same procedure was applied until all mice were able to stay onto the platform for 15 s (usually three to four trials).

#### Sensorimotor Testing

The next day sensory motor abilities of all mice were tested during three trials with a 60 s cut-off and a 5 min inter-trial interval (ITI) using a visible cued platform located in the northwest (NW) quadrant of the pool. A trial terminated when the animal climbed onto and stayed on the platform for 15 s. Mice that failed to find the platform within a 60 s cut-off time were placed on it for 15 s by the experimenter. Between each trial, mice were held in their home cages. For both pre-training and sensorimotor testing, the pool was surrounded by a black curtain with no cues. For the rest of the experiment, the curtain was removed and mice had access to the distal cues located in the room.

#### Training with Variable Start Positions

On the day following sensorimotor testing, mice were trained for three daily trials with a cut-off of 60 s and 5 min ITI to find the platform located in the northeast (NE) quadrant. Mice were released from three different starting points at each trial, and different sequences of starting points were used day by day. The training lasted 11 days.

#### Reversal

Thirteen days after the last session, mice were trained in a reversal protocol whereby they had to find the new location of the platform that was moved to the northwest (NW) quadrant. Their performances were analyzed over 4 days using three daily trials with a 5 min ITI.

#### Training in Middle Aged Mice

Eight months later, mice were tested again in the same task using the same procedures (two WT and one heterozygous mice had to be eliminated for inconsistent behavior): they were first exposed to 10 days of training to find the hidden platform located in the NW quadrant, upon which a probe test was performed to measure memory of the platform location; to this end, the hidden platform was removed from the pool and each subject was placed into the water diagonally opposite to the target quadrant. Time spent in the target quadrant (% of total time, chance level = 25%) was measured over 60 s.

Two days later their ability to locate the same platform moved to the NE quadrant was also assessed over 4 days in a reversal test. Twenty-four hours later, a probe test was performed.

### Statistical Analyses

All statistical analyses were performed with Statistica 12.0 software (Statsoft). Neurogenesis and anxiety-related data were analyzed with Student’s *t-*tests, while water maze data were analyzed with two-way ANOVAs using NK *post-hoc* test whenever appropriate. All data are presented as mean ± SEM.

## Results

### Vangl2^Lp^ Mutation Affects the Survival of Adult-Born Neurons

First, we investigated the impact of the Looptail mutation on the number of cells immunoreactive for doublecortin (Figure 1B), an immature neuronal marker used as a surrogate of neurogenesis. We found a decreased number of DCX-IR cells in both the dorsal (*t*_14_ = −3.53, *p* = 0.003) and ventral dentate gyrus (DG; *t*_14_ = −2.67, *p* = 0.01) resulting in an overall decrease (*t*_14_ = −4.14, *p* = 0.0009) and indicating that looptail mutation decreases the number of cells engaged in a neuronal lineage. We analyzed the dendritic morphology of these newborn cells and performed Sholl analyses on DCX-IR cells with tertiary dendrites. We found that neither cell body area (*t*_72_ = −0.56, *p* = 0.57), number of branching points (*t*_72_ = −0.62, *p* = 0.53), dendritic length (*t*_72_ = 0.58, *p* = 0.56), or distribution of dendrites along the dendritic tree (Figure 1C; genotype effect *F*_1,71_ = 0.09, *p* = 0.76; genotype × distance from soma interaction *F*_24,1704_ = 0.67 *p* = 0.87) were influenced by the Vangl2^Lp^ mutation. This was true also when we separated the analysis of DCX-IR cells located into the dorsal and ventral parts of the DG (*dorsal part*: cell body area *t*_39_ = −0.59, *p* = 0.55, number of branching points *t*_39_ = −0.68, *p* = 0.49, dendritic length *t*_39_ = 1.15, *p* = 0.25, distribution of dendrites along the dendritic tree genotype effect *F*_1,39_ = 0.034, *p* = 0.85; genotype × distance from soma interaction *F*_22,836_ = 0.87 *p* = 0.62; *ventral part*: cell body area *t*_31_ = −0.20, *p* = 0.83, number of branching points *t*_31_ = −0.25, *p* = 0.79, dendritic length *t*_31_ = −0.58, *p* = 0.56, distribution of dendrites along the dendritic tree genotype effect *F*_1,31_ = 0.34, *p* = 0.56; genotype × distance from soma interaction *F*_22,682_ = 0.90 *p* = 0.58). In order to determine whether the reduction in DCX-IR cells was linked to a deficit in cell proliferation and/or a deficit in cell survival, we first labeled dividing cells using Ki67 and phosphorylated-Histone 3 (pH3). We found no differences in the number of cells immunoreactive for Ki67 or pH3 between WT and *Vangl2^Lp/+^* mice whether in the dorsal (*t*_13_ = −1.18 *p* = 0.25; *t*_12_ = −0.71 *p* = 0.48, for Ki67 and pH3, respectively), ventral (*t*_13_ = 0.53 *p* = 0.60; *t*_12_ = 0.79 *p* = 0.44, for Ki67 and pH3, respectively), or total DG (*t*_13_ = −0.66 *p* = 0.52; *t*_12_ = −0.12 *p* = 0.90, for Ki67 and pH3, respectively), indicating that looptail mutation does not affect cell proliferation (Figures 1D,E). To analyze cell survival, mice were injected with the thymidine analogs IdU and CldU at 2 weeks interval and the number of surviving cells was counted 2 weeks after the last injection (Figure 1A). Two-week-old CldU-IR cell number (Figure 1F) was reduced in *Vangl2^Lp/+^* mice compared to WT (*t*_14_ = −2.06, *p* = 0.05), with a contribution of both the dorsal and ventral parts, although differences did not reach significance (*t*_14_ = −1.99 *p* = 0.06 and *t*_14_ = −1.49 *p* = 0.15 for dorsal and ventral DG, respectively). This reduced survival of newly-born cells was confirmed in 4 weeks old IdU-IR cells (Figure 1G; *t*_13_ = −3.97, *p* = 0.001) in both the dorsal (*t*_13_ = −4.11, *p* = 0.001) and ventral DG (*t*_13_ = −2.84, *p* = 0.01). This was not accompanied by any modification in the volume of the granule cell layer (*t*_14_ = −0.72, *p* = 0.47). If altogether these data indicate an involvement of Vangl2 in cell survival, the larger effect observed in IdU-IR cells suggests a mechanism targeting preferentially 2–4 weeks old cells. We thus analyzed whether the fate of these surviving cells was affected by the looptail mutation (Figures 1H,I) and found no differences in the percentage of 4-week- old cells acquiring a neuronal (IdU-Calbindin-IR cells, *t*_12_ = 0.80, *p* = 0.43) or a glial phenotype (IdU-S100-IR cells, *t*_12_ = −0.64, *p* = 0.52). Together with the reduced number of IdU-IR cells, these data indicate a decreased production of newborn neurons in *Vangl2^Lp/+^* mice and support a role for Vangl2 in adult-born cell survival.

To determine whether this role is specific to the DG, we measured cell survival in the OB and found no difference in the volume of the GCL (*t*_13_ = −0.72, *p* = 0.47), or the number of 4-week-old IdU–IR cells (WT *n* = 65545+/− 5104, *Vangl2^Lp/+^* *n* = 61651+/− 1916; *t*_13_ = −0.75, *p* = 0.46) between WT and *Vangl2^Lp/+^* mice. Although these results do not preclude a specific effect on other parameters of adult neurogenesis, such as neuroblasts migration or neuronal morphology, which were found to be altered in the OB after altering Vangl2 expression using a knock-down (Vangl-2KD) or a dominant-negative form (Vangl2-DN; Hirota et al., 2012), they nevertheless indicate that Vangl2 involvement in neuronal survival is specific to the hippocampal neurogenic niche.

### Vangl2^lp^ Mutation Alters Memory Flexibility but Not Anxiety-Related Behavior in Adult Mice

We next sought to investigate the consequences of these neurogenesis deficits on mice abilities to navigate through space. Because of their phenotype (looptail), we first tested whether mutant mice were impaired in their abilities to swim toward a goal and exposed them to three trials with a cued platform (Figure 2A). Although slightly less rapid than WT mice (mean swim speed WT = 21.81 ± 0.34 cm.s^−1^, *Vangl2^Lp/+^* = 18.03 ± 0.7 cm.s^−1^; genotype effect *F*_1,30_ = 27.98, *p* < 0.0001) heterozygous mice did not significantly differ in their latency (Figure 2B; genotype effect *F*_1,30_ = 0.23, *p* = 0.63; genotype × trial interaction *F*_2,60_ = 0.34, *p* = 0.71) or distance (Figure 2C; genotype effect *F*_1,30_ = 2.61, *p* = 0.11; genotype × trial interaction *F*_2,60_ = 1.42, *p* = 0.25) to reach the platform over the three trials. Then animals were tested for their ability to find a hidden platform (the position of which remained unchanged during training) from variable starting positions. This type of memory, i.e., reference memory was not affected either and both latency (Figure 2D) and distance (Figure 2E) to find the platform decreased similarly in *Vangl2^Lp/+^* and WT mice over days of training (latency: genotype effect *F*_1,30_ = 0.35, *p* = 0.55; genotype × training day interaction *F*_10,300_ = 0.48, *p* = 0.89; distance: genotype effect *F*_1,30_ = 0.04, *p* = 0.83; genotype × training day interaction *F*_10,300_ = 0.68, *p* = 0.74).

Given that we and others have shown that new neurons are involved in cognitive flexibility (Dupret et al., 2008; Garthe et al., 2009), mice were then tested in a reversal version of the test: the position of the platform was changed and the ability of mice to learn the new position (and forget the old one) was measured. This procedure revealed major deficits in *Vangl2^Lp/+^* mice, with an increase in both latency (Figure 2F) and distance (Figure 2G) to find the new platform location, although the difference did not reach significance for distance (latency: genotype effect *F*_1,30_ = 5.16, *p* = 0.03; genotype × training day interaction *F*_3,90_ = 2.06, *p* = 0.11; distance: genotype effect *F*_1,30_ = 3.14, *p* = 0.08; genotype × training day interaction *F*_3,90_ = 2.41, *p* = 0.07). When the analysis was performed over the last 3 days of training, which reflect the actual learning phase of the new platform location, both latency and distance were significantly altered in *Vangl2^Lp/+^* mice (latency: genotype effect *F*_1,30_ = 7.06, *p* = 0.01; genotype × training day interaction *F*_2,60_ = 0.16, *p* = 0.84; distance: genotype effect *F*_1,30_ = 5.37, *p* = 0.02; genotype × training day interaction *F*_2,60_ = 0.25, *p* = 0.78) reflecting a failure to adapt to the changed situation and to develop appropriate new spatial preference in 4 month-old mice.

In light of neurogenesis’ involvement in anxiety (Revest et al., 2009), we next tested the emotional status of *Vangl2^Lp/+^* mice in two tests aimed at measuring their anxiety level. In the elevated plus maze where anxious mice usually avoid open arms, WT and heterozygous mice spent a similar amount of time in these arms (Figure 3A; *t*_35_ = −0.97, *p* = 0.33) and showed the same level of general exploration (total distance traveled: Figure 3B, *t*_35_ = 0.27, *p* = 0.78; total number of visited arms: Figure 3C, *t*_35_ = 0.70, *p* = 0.48). When given the choice between an aversive brightly lit and open environment and a typically preferred dark and enclosed one in the Light/Dark test, mice from both genotypes took the same time to engage in exploring the lit environment (Figure 3D, *t*_35_ = −0.27, *p* = 0.78), made the same number of transitions between the two compartments (Figure 3E, *t*_35_ = 0.02, *p* = 0.97) and spent a similar amount of time in the enclosed reassuring environment (Figure 3F, *t*_35_ = 0.34, *p* = 0.73). Together these results demonstrate that the looptail mutation is not a sufficient condition to impact the anxiety level of mice.

### Vangl2^Lp^ Mutation Alters Capability to Handle Memory Interferences in Middle Aged Mice

We next questioned whether the deficit in flexibility observed in adult mice would persist and whether memory abilities worsen with age. At 12 months of age, the same mice were retested in the water maze (Figure 4A). Twelve months old WT mice learned more quickly the task compared to 4 months old (age effect on latency: *F*_1,16_ = 38.56, *p* < 0.001), while *Vangl2^Lp/+^* mice showed the same rate of learning (age effect on latency: *F*_1,10_ = 2.76, *p* = 0.12) indicating that they were not sensitive to the beneficial effect of retraining (genotype × age interaction on latency: *F*_1,26_ = 4.56, *p* = 0.04; WT 12-month < WT 4-month at *p* < 0.001; Vangl2 ^Lp/+^ 12-month = 4-month). As a result, analysis of learning curves in 12 months old mice revealed a delay in learning platform location over days in *Vangl2^Lp/+^* mice compared to WT as revealed by differences in latency (Figure 4B, genotype effect *F*_1,27_ = 9.15, *p* = 0.005; genotype × training day interaction *F*_9,243_ = 0.61, *p* = 0.78). Learning was then divided into two phases: an early phase (first 5 days) during which there is a fast and large improvement in performance and a late phase (last 5 days) during which performances remain at a stable level (Dobrossy et al., 2003). We found that *Vangl2^Lp/+^* mice were impaired during the last phase of learning, indicating deficits in memory stabilization (latency: genotype effect *F*_1, 27_ = 13.36, *p* = 0.001; genotype × training day interaction *F*_4,108_ = 0.54, *p* = 0.70; distance: genotype effect *F*_1,27_ = 8.01, *p* = 0.008; genotype × training day interaction *F*_4,108_ = 0.24, *p* = 0.91). This delay did not affect their memory for platform location tested 24 h after the last training trial in a probe test as mice from both genotypes spent more time in the target quadrant (TQ, NorthWest position) than in any other one (WT: quadrant effect *F*_3,51_ = 25.82, *p* < 0.0001; TQ > each other quadrant at *p* < 0.001; *Vangl2^Lp/+^*: quadrant effect *F*_3,30_ = 14.43, *p* < 0.0001; TQ > each other quadrant at *p* < 0.001). We next carried out a reversal test and found that as in their young age, middle aged heterozygous mice were impaired in their rate of learning as both latency and distance to reach the platform were increased compared to WT (latency: Figure 4E, genotype effect *F*_1,27_ = 11.26 *p* = 0.002; genotype × training day interaction *F*_3,81_ = 0.18 *p* = 0.90; distance: Figure 4F, genotype effect *F*_1,27_ = 4.49 *p* = 0.04; genotype × training day interaction *F*_3,81_ = 0.13 *p* = 0.93). A probe test performed after 4 days of training indicated that while WT mice acquired the new platform location and spent significantly more time in the new target quadrant (new TQ) than in any other one (quadrant effect: *F*_3,51_ = 19.12, *p* < 0.0001; new TQ > each other quadrant at *p* < 0.01), *Vangl2^Lp/+^* mice persevered swimming to the previous platform position (quadrant effect *F*_3,30_ = 10.66, *p* < 0.0001, new TQ = old TQ > SE and SW at *p* < 0.05) confirming deficit in cognitive flexibility. Altogether these data indicate that Vangl2 may be involved in protecting cognitive capabilities from age-related decay.

## Discussion

In this study, we investigated the involvement of the core PCP gene *Vangl2* in adult hippocampal neurogenesis and spatial memory abilities in the course of aging. We report that heterozygote Lp mutation is sufficient to impair the survival of adult hippocampal newborn neurons and alter cognitive flexibility in adult mice. In addition, it accelerates the age-related deterioration of episodic-like memory by increasing susceptibility to interference, leading to deficits in spatial navigation. Altogether these results demonstrate for the first time a key role for members of the Wnt/PCP pathway in regulating neurogenesis-dependent episodic-like memory in the course of aging.

### Looptail Mutation Regulates the Number of Adult-Born Neurons

Although the PCP pathway has been extensively studied in embryonic development, the consequences of its defects in adults are poorly understood. Given the known role of this pathway in actin cytoskeleton remodeling and cell migration, we hypothesized that the PCP pathway might be important for several aspects of adult neurogenesis. We indeed report a decrease in the number of newly-born neurons resulting from a diminution of the survival of 2- to 4-week old newborn cells, without modifications in cell proliferation. This result is in line with the few studies that have analyzed the involvement of PCP components on hippocampal adult neurogenesis. Thus Wnt-5a, which directly regulates the level of Vangl2 phosphorylation through Ror2, a step required for Vangl2 function in PCP during mammalian development (Gao et al., 2011; Yang et al., 2017), reduces the number of DCX-IR cells and the number of newborn neurons reaching maturity without modifying cell proliferation (Arredondo et al., 2020). The lack of effect on cell fate determination is also consistent with the consequences of knocking down FZD3 or Celsr1–3, other core PCP components (Schafer et al., 2015). Although the overall data available are thus still too sparse to draw a mechanistic hypothesis, our observations may result from both a direct effect of Vangl2 altered expression in DCX-IR cells and an indirect effect of disturbances in the cellular niche that encompasses developing newborn neurons. Interestingly, whatever the involved mechanisms, neurogenesis in the OB was spared, indicating a different role of Vangl2 in the two adult neurogenic niches.

These effects are clearly distinct from those reported for the canonical members of the Wnt pathway, which have been proposed to be important for regulating the balance between maintenance of the stem cell pool and differentiation of newborn neurons (Wexler et al., 2009; Varela-Nallar and Inestrosa, 2013) based on the following evidences: (i) general blockade of Wnt signaling in the DG reduces proliferation and neurogenesis while its activation increases neurogenesis (Lie et al., 2005); (ii) the Wnt signaling inhibitors Dickkopf 1 (Dkk1) and secreted Frizzled-related protein 3 (sFRP3) negatively regulate neurogenesis (Jang et al., 2013; Seib et al., 2013; Sun et al., 2015); (iii) Frizzled 1 (FZD1), a well-known receptor for the Wnt/β-catenin pathway, controls neuronal differentiation and newborn cells migration (Mardones et al., 2016); (iv) manipulation of GSK-3β levels, a key player in Wnt/β-catenin activation, affects proliferation and neural fate specification (Eom and Jope, 2009; Fiorentini et al., 2010). Together with our data, this suggests a complementary role for canonical and PCP Wnt components in regulating different aspects of adult neurogenesis.

Because one of the main targets of core PCP signaling is the modulation of the cytoskeleton (Babayeva et al., 2011), we expected that Vangl2 would regulate the branching of dendritic trees in newborn neurons. This would have been in line with recent studies in which Vangl2 knockdown (Vangl2KD), Vangl2null mutation (Vangl2NM) and looptail mutation (Vangl2^Lp/+^) led to a global decrease in the complexity of dendritic branching and the number of spines in dissociated hippocampal and cortical neurons (Hagiwara et al., 2014; Okerlund et al., 2016). In accordance with these results obtained in cultured cells, other *in vivo* studies reported a similar reduction in dendritic tree complexity and spine density specifically in adult-born granule cells after disruption of other PCP genes such as Wnt-5a, FZD3, or Celsr2/3 (but not Celsr1) in the adult DG (Schafer et al., 2015; Arredondo et al., 2020). However, these results are contrasted by a recent study using Vangl2 conditional deletion (Vangl2cKO) in which an increase in spine density of CA1 hippocampal pyramidal cells and an opposite role for Vangl2 and Celsr3 were reported (Thakar et al., 2017). Altogether, these data suggest a complex role of Vangl2 in regulating neuronal morphology and spinogenesis that has been further emphasized in our recent *in vivo* study showing that postnatal deletion of Vangl2 (Vangl2cKO) leads to different alterations of the dendritic arbor and spine density of Golgi-stained dentate granule neurons (DGNs) depending on the distance from the soma, with decreased complexity in the proximal section and increased ramification in the more distal one (Robert et al., 2020). Interestingly, we also reported in this study a dual distribution of Vangl2 protein during the maturation period of granule cells, with an enrichment in the cell bodies of immature postmitotic DGNs and massive redistribution to the neurites of mature DGNs (Robert et al., 2020). This may lead to an enrichment of Vangl2 in the cell bodies of DCX-IR cells that could explain why dendritic branching was not affected in our targeted population, although differences in experimental approaches and models (Vangl2Lp vs. Vangl2cKO) may also certainly explain these divergent results.

### Looptail Mutation Accelerates Age-Related Memory Disorders

Adult neurogenesis has been widely associated with anxiety-related behaviors and spatial learning and memory capabilities (Snyder and Drew, 2020; Abrous et al., 2021) so we analyzed the consequences of *Vangl2* mutation on both affective and cognitive functions. Contrary to our expectations in light of their reduced neurogenesis, we did not evidence any deficit in measures of anxiety in looptail mice. Although unexpected, the same result was found after conditional deletion of Celsr3, another core PCP component (Thakar et al., 2017), or more recently in our study using conditional deletion of Vangl2 in postmitotic neurons (Robert et al., 2020). This absence of a relationship between neurogenesis reduction and anxiety levels is not uncommon and for instance, exercise, which strongly enhances neurogenesis (Vivar and van Praag, 2017), was found to both increase (Burghardt et al., 2004; Fuss et al., 2010) and decrease anxiety (Salam et al., 2009; Llorens-Martín et al., 2010; Tomiga et al., 2021). On the same line, mouse models of suppressed hippocampal neurogenesis report similar discrepancies, with some studies observing no link (Saxe et al., 2006) while others show heightened (Bergami et al., 2008; Revest et al., 2009) or reduced (Uchida et al., 2011) anxiety in conjunction with reduced neurogenesis. This suggests a complex relationship dependent on rodent strain and model, housing conditions, timing and nature of tests employed, and most of all the stress state of the animal (Petrik et al., 2012; Hill et al., 2015; Yun et al., 2016).

On the other hand, consistent with our previous reports linking alterations in adult neurogenesis with deficits in memory processes (Dupret et al., 2008; Tronel et al., 2012) we found that adult mutant mice were impaired in the water-maze task solving. Indeed, although their basic spatial navigation learning abilities were spared, which is consistent with what we previously reported after genetic ablation of *Vangl2* in postmitotic neurons (Robert et al., 2020), their performances in a reversal version of the task were altered, and they showed a delay in reacquisition following goal reversal. As this task requires a high degree of flexibility since mice have to transfer previously learned relationships between environmental spatial cues to the novel situation (i.e., changed goal position), results in adult looptail mice are indicative of a deficit in behavioral flexibility. This deficit was also evident when mice were retested at middle age, indicating that the early behavioral deficit linked to *Vangl2* mutation is consistent with an advanced onset of decrease in flexibility otherwise observed with normal aging in mice (Matzel et al., 2011; Yang et al., 2019), rats (Mota et al., 2019), non-human primates (Joly et al., 2014) and humans (van Boxtel et al., 1998). It is also interesting to highlight that although repeated training improved performances in control mice, mutant mice did not benefit from this previous training suggesting that they did not remember the task. In fact memory deficits worsened with age as middle-aged mutant mice were impaired during both the initial learning phase and during reversal. When a probe test was performed at the end of the reversal, mutant mice persevered swimming to the previous platform position confirming deficits in cognitive flexibility that may result from their inability to erase old irrelevant information to the benefit of new relevant ones; such inability is highly suggestive of alterations in the interference-based form of active forgetting (Davis and Zhong, 2017), although we cannot exclude that alterations of retrieval-induced forgetting and/or intrinsic forgetting can also be at play.

From a mechanistic point of view, although our data do not directly demonstrate the involvement of adult neurogenesis in the behavioral deficits exhibited by Vangl2 deficient mice, they are consistent with the putative role of adult-born neurons in relational memory, flexibility, and forgetting. Indeed, we and others have previously uncovered the specific role of adult-born neurons in supporting behavioral flexibility (Dupret et al., 2008; Garthe et al., 2009; Burghardt et al., 2012), and several recent studies strongly support their involvement in the balance between forgetting previously-acquired memories and learning new information that conflicts with these previously acquired memories, a process also known as active forgetting (Akers et al., 2014; Epp et al., 2016; Tran et al., 2019). In particular, computational and experimental models taken together indicate that decreasing neurogenesis stabilizes existing memories, thereby enhancing interference for the encoding of new memories, particularly when existing and new memories overlap in content, as is the case with goal reversal in the water maze (Aimone et al., 2009; Epp et al., 2016). As a consequence, this impedes the encoding of the new, conflicting information, as we observed in mutant mice. While such stabilization may hold an adaptive value under physiological conditions, in the present case, it reflects an inability to adjust to the changing environment and thus a lack of flexibility.

Although studies tackling the behavioral consequences of manipulating Wnt/PCP genes are scarce, the present results may at first sight seem contradictory to our recent study where deletion of *Vangl2* in postmitotic neurons of the DG led to improved pattern separation abilities (Robert et al., 2020). Indeed, pattern separation was found to serve cognitive flexibility by reducing interference between closely related information sets (Wiskott et al., 2006), and we would thus expect alterations in pattern separation abilities when altering Vangl2 levels. However, from a mechanistic point of view, because newborn neurons have been implicated in reducing interference between closely related information sets by heightened likelihood of activation in response to novel cues and dampening activation patterns associated with closely related past experiences (Danielson et al., 2016; Drew et al., 2016), the decreased neurogenesis seen in *Vangl2^Lp/+^* mice is consistent with their increased susceptibility to interference. When looking at consequences of postmitotic deletion of *Vangl2*, no such reduction in neurogenesis was observed and on the contrary, an increase in the number of newly-generated immature neurons, which are known to sustain pattern separation abilities (Clelland et al., 2009; Aimone et al., 2011; Sahay et al., 2011; Tronel et al., 2012), was recorded (Robert et al., 2020). The two models are thus highly complementary in solving the exact contribution of Vangl2 to the behavioral consequences of altered PCP signaling and understanding the mechanisms involved.

Finally, our result also supports the idea that the decreased neurogenesis observed from early adult life in mutant mice is involved in the accelerated aging of their cognitive functions. Indeed, we and others have previously shown that adult neurogenesis is one of the core mechanisms in the aging of memory functions (Drapeau and Abrous, 2008; McAvoy and Sahay, 2017). For instance, we have shown that increasing and decreasing adult neurogenesis through modulation of the HPA axis activity protects and prevents, respectively, from the appearance of memory disorders measured in the Water-maze from middle age onwards (Lemaire et al., 2000; Montaron et al., 2006). Using more specific approaches, recent studies have confirmed this link, showing that enhancement of adult-born DGCs integration by genetic overexpression of Kruppel-like factor 9, a negative regulator of dendritic spines, in mature dentate granule neurons (McAvoy et al., 2016) or enhancement of neural stem cells expansion by overexpression of the cell cycle regulators Cdk4/cyclinD1 (Berdugo-Vega et al., 2020) improve memory abilities of middle-aged mice.

In summary, the results presented here show for the first time that a single allele deregulation of Vangl2 is sufficient to impair adult hippocampal neurogenesis and cognitive flexibility when animals are young adults and to accelerate the age-related decline in spatial learning most probably through an alteration of interferences-induced forgetting. These results pinpoint that Vangl2 activity could be a predictive factor of successful aging and open tantalizing opportunities for the prevention of age-related cognitive deficits.

## Data Availability Statement

The raw data supporting the conclusions of this article will be made available by the authors, without undue reservation.

## Ethics Statement

The animal study was reviewed and approved by Animal Care Committee of Bordeaux CEEA50.

## Author Contributions

MK and DNA conceived experiments. MK and EL performed experiments. MK analyzed data and wrote original draft. MK, MM, and DNA reviewed and edited manuscript. MM provided biological resources. DNA provided funding. All authors contributed to the article and approved the submitted version.

## Conflict of Interest

The authors declare that the research was conducted in the absence of any commercial or financial relationships that could be construed as a potential conflict of interest.

## Publisher’s Note

All claims expressed in this article are solely those of the authors and do not necessarily represent those of their affiliated organizations, or those of the publisher, the editors and the reviewers. Any product that may be evaluated in this article, or claim that may be made by its manufacturer, is not guaranteed or endorsed by the publisher.
